# Effects of short-distance transportation on physiological indexes, intestinal morphology, microbial community, and the transcriptome of the jejunum in weaned piglets

**DOI:** 10.3389/fvets.2023.1148941

**Published:** 2023-04-12

**Authors:** Qin Fu, Xuesong Yang, Sitong Zhou, Yue Yang, Xiaohong Zhang, Qi Han, Wenbo Ji, Honggui Liu

**Affiliations:** ^1^College of Animal Science and Technology, Northeast Agricultural University, Harbin, Heilongjiang, China; ^2^Key Laboratory of Swine Facilities Engineering, Ministry of Agriculture and Rural Affairs, Harbin, Heilongjiang, China

**Keywords:** inflammation, 16S rRNA sequencing, microbial community, transcriptome, weaned pig

## Abstract

Transportation of livestock is unavoidable in animal production. A total of 72 piglets were randomly divided into the CON group and the TSG group, and the piglets in CON group were transported for two hours. The purpose of this study was to determine the effects of short-distance road transportation lasting 2 h on the jejunum of weaned piglets. Our results showed that compared with the control group, there was no impact on the growth performance of piglets in the transport group (*P* > 0.05). The concentrations of cortisol, heat shock protein (HSP)70, HSP90, C-reactive protein, interleukin (IL)-6, IL-8, IL-12, and interferon-γ and the activity of reactive oxygen species were increased in the jejunum of piglets in the transport group (*P* < 0.05 compared with the control group). The concentrations of glutathione peroxidase, claudin-1, occludin, and zonula occludens-1 showed no between-group differences (*P* > 0.05). Regarding intestinal morphology, the transport group showed infiltration of a small amount of lymphocytes into the jejunum mucosa epithelium that was accompanied by edema of the lamina propria, whereas the control group showed no obvious abnormalities. At the genus level, in the transport group, the 16S rRNA sequencing revealed a downward trend in the relative abundance of *Lactobacillus* and an upward trend in the relative abundance of *Muribaculaceae*_*unclassified*. There was also increased mRNA expression of genes associated with inflammation in the transport group, but the genes and pathways related to apoptosis were not activated. In summary, weaned piglets undergoing 2 h of short-distance road transportation showed stress and inflammatory reactions of the jejunum but did not exhibit oxidative damage or activation of the apoptosis pathway of the jejunum. Furthermore, the growth performance of the piglets was not affected by the trip.

## Introduction

Transportation is an unavoidable process in animal husbandry and production (1). Piglets are mainly transported to separate production facilities at the time of weaning to reduce the vertical transmission of disease, provide special care, and improve the early growth, health and welfare, and production potential of weaned pigs. Transportation stressors in piglets include loading conditions, ambient temperature, loading density, handling, unfamiliar smells, noise, vibration, and sudden speed changes (2, 3). Such stressors can lead to physiological and behavioral changes in pigs, especially in young animals (4). These changes include increases in fighting behavior, dehydration, cortisol levels, and acid-base imbalance in piglets (4–6).

The influence of road transportation on piglet welfare is considered to be an under-represented research field (2, 7), particularly regarding its potential effect on the piglet intestine. Indeed, stressful conditions in young pigs have been shown to lead to intestinal mucosal congestion, intestinal villi injury, intestinal barrier damage, intestinal immune system activation, and intestinal inflammation promotion (8). The increased secretion of inflammatory factors impacts the endocrine system as well as the nervous system through blood circulation, thus affecting the overall state of stress (9). Although stress is understood to easily induce inflammation, there is little research on the effects of transportation stress on intestinal inflammation.

It has been shown that the level of reactive oxygen species (ROS) rises sharply during transportation, and an imbalance between the production and elimination of these molecules may lead to oxidative stress (10). High concentrations of ROS attack the carbohydrates, lipids, proteins, and nucleic acids of the intestinal endothelial cells, leading to oxidative damage of these cells and affecting the structure and function of the intestinal tract (11–13). Studies by Perry et al. and Zou et al. showed that stress from long-distance travel can also adversely affect the gut microflora and structure, leading to oxidative stress and inflammation (14, 15). These conditions may lead to intestinal mucosal barrier damage and dysfunction, which promote the occurrence of bacterial infection and infectious diseases.

With the sale and transfer of piglets to fattening housing becoming more common, the short-distance transportation of piglets has become an important link in animal husbandry and production. In one study, only 1 h of road travel caused physiological and metabolic imbalances in piglets (4). Intestines are easily damaged by stress. Furthermore, the intestinal tract is an essential place for the digestion, absorption, and metabolism of nutrients (16), all of which affect the production of piglets. Therefore, the potentially harmful effects of short-distance transportation on piglets, including the degree of damage to their intestines, have aroused public health concerns. The intestinal tract is the largest organ of digestion, absorption, and immunity in the body, and the jejunum plays a major role in digestion and immunity in the small intestine. Therefore, we chose the jejunum as the research object. The purposes of this study were to explore the influence of short-distance road transportation on the piglet intestine through the detection of physiological indexes, microbial sequencing, and transcriptome determination and to determine whether it has a negative impact on the production performance of piglets.

## Materials and methods

All experiments and procedures carried out in this study were approved by the Animal Protection and Utilization Committee of Northeast Agricultural University (NEAUEC20200346) and were conducted under the guidelines of the “Rules of Animal Protection and Utilization of Northeast Agricultural University”.

### Piglets and housing

Our experimental animals were crossbred (Large White × Landrace) weaned pigs obtained from the Northeast Agricultural University experimental farm in Harbin, China. Piglets from this farm began receiving creep feed at 20 days of age, were weaned at 28 days, and were gradually administered a nursery diet. Complete feeding with the nursery diet was started at 40 days of age. The nutritional components of the creep feed and nursing diet are shown in Supplementary material S1. At the age of 42 days, a total of 72 piglets with an initial body weight of 11.82 ± 0.04 kg were selected and randomly divided into the control group (CON) and transport stress group (TSG), with 36 pigs in each group. There were six replicate pens per group and six pigs per pen (1.58 × 1.71 m). The piglets in each pen were marked with numbers and different colors of paint. The two groups were raised under the same conditions for 1 week, during which all piglets had free access to feed and water. The road transport experiment was undertaken at 49 days of age. At 9 pm on the day before transport, food (not water) was withdrawn from both groups of piglets. The average food intake of all piglets on the pre-transport day was about 0.67 kg/piglet. During the transportation of the TSG group of piglets, consistent with the TSG group, the piglets in the CON group were not provided with food and water.

### Transportation phase

Before transport, we disinfected the specially designed transport truck, which featured three floors, each with a capacity to hold six separate fenced pens (length × width: 210 × 110 cm) and comprising an area of about 2.31 m^2^. All three floors, each containing 12 TSG group piglets divided into two pens (transportation density, ~0.19 m^2^/piglet), were used for the experiment. The metal flooring was covered with a 2-cm-thick straw layer. No water or feed was provided at any point in the transport, during which the average outdoor temperature ranged from 24°C to 28°C, and the average humidity was 41.5%. The piglets were transported in the truck by an experienced driver, along a flat road, for a relatively short distance (120 km) for 2 h (average speed, 60 miles/h) before returning to the starting point.

### Sample collection and body weight recording

After the transport, the TSG group piglets were unloaded from the truck and weighed individually. Subsequently, six of these piglets (one from each pen) were selected for euthanasia by electric shock (220 V, 1.25 A for 3 s). Similarly, the piglets in the CON group were also weighed separately before selecting six piglets for euthanasia (220 V, 1.25 A for 3 s). Immediately, the jejunum was quickly isolated, transferred to an ice bath, and sheared. After trimming 5 cm near the proximal end, we collected jejunal tissue segments of about 4 cm in length. A portion of the segment was cut into smaller (1 cm^3^) pieces, transferred to a tube containing 4% paraformaldehyde, and stored at 4°C for morphological analysis. Samples from the middle segment of the jejunum were transferred to RNase-free Eppendorf tubes and stored at −80°C for determination of physiological indexes, gene expression, and transcriptome analysis. Finally, under sterile conditions, the fecal contents of the jejunum were collected, transferred to RNase-free freezing tubes, labeled, and quickly frozen in liquid nitrogen. After returning to the laboratory, the fecal samples were stored at −80°C until testing. Both groups of piglets were returned to the previous feeding mode for 2 weeks and were then re-weighed (age, 63 days). The bodyweight records are shown in Table 1.

**Table 1:** Effect of short-distance transportation on the performance of piglets^a^.

**Indexes**	**CON**	**TSG**	**SEM**	***p*-value**
49-day body weight, kg	14.99	14.95	0.051	0.857^ns^
63-day body weight, kg	22.27	22.16	0.627	0.759^ns^
Average daily weight gain in 49-63 days, kg	0.520	0.515	0.0059	0.681^ns^

a The values represent the means of 6 pens, with 6 piglets in each repeated pen (*n* = 36) for body weight and 6 pens in each group (*n* = 6).

SEM, Standard Error of Mean; *p*-value, the *p*-value.

ns Represented no significant difference, *P* > 0.05.

### Determination of physiological indexes in jejunal samples

The jejunal tissue (0.5 g) was rinsed in precooled normal saline to remove residual blood, dried with filter paper, weighed, and transferred to a small (10 mL) beaker. Precooled 0.86% normal saline (9 times the weight of the tissue) was added to the beaker, and then, the tissue was cut into smaller pieces and homogenized with a hand-held homogenizer. The prepared homogenate was centrifuged for 10 min at 2,000 r/min, and the supernatant was collected and used to determine the concentrations of the following: cortisol, heat shock protein (HSP)70, HSP90, glutathione peroxidase (GSH-PX), C-reactive protein (CRP), interleukin (IL)-6, interleukin (IL)-8, IL-12, interferon (IFN)-γ, and three tight junction proteins [Claudin-1, Occludin, zonula occludens-1 (ZO-1)] (ELISA kit, Nanjing Jiancheng Bioengineering Institute, Nanjing, China). The amount of protein in the homogenate was determined by a Coomassie blue assay kit (Nanjing Jiancheng Bioengineering Institute, Nanjing, China). To determine ROS activity, a separate jejunal tissue sample (0.5 g) was washed with precooled phosphate-buffered saline (PBS) to remove residual blood and then cut into pieces. A corresponding volume of PBS buffer (nine times the weight of the tissue) was added to the tissue, followed by an appropriate amount of digestive enzyme, and the mixture was incubated at 37°C for 30 min. Digestion was terminated by adding PBS. The mixture was filtered, centrifuged at 500 × g for 10 min, and the precipitate was collected for ROS determination by biochemical kit (Nanjing Jiancheng Bioengineering Institute, Nanjing, China), following the manufacturer's instructions. Finally, read the number of ROS with an enzyme-labeled instrument.

### Western blot detection

The jejunum tissue was lysed with western blot and immunoprecipitation (IP) lysis buffers (P0013, Biosharp, China) containing protease inhibitors (Beyotime, China). According to the instructions of the kit, the protein concentration of all samples was determined by the BCA protein determination kit (Beyotime, China). Initially, 20 μg of lysate was electrophoresed through 10% or 12% SDS-PAGE gels (Solarbio, China). Then, proteins were transferred to polyvinylidene fluoride (PVDF) membranes (Cytiva, Marlborough, MA, USA). At room temperature, the membrane was blocked with 5% BSA for 1 h and incubated with the first antibody at 4°C overnight and with the second antibody against rabbit IgG (1:2000, Santa Cruz, USA) for 1 h at room temperature. The HSP 70 (HSP70, 1:800) and HSP 90 (HSP 90, 1:1000) were detected, and β-actin (1:5000) was used as the analysis reference. BOX Chemi XX9 imager (Syngene, Cambridge, UK) was used to detect protein signals with potentiated chemiluminescence (ECL) reagents (Beyotime, China). ImageJ software (National Institutes of Health, Bethesda, MD) was used to quantify the relative density of bands.

### Detection of intestinal morphology

Jejunal tissue samples fixed in paraformaldehyde were dehydrated with ethanol, which was gradually replaced with xylene, and then embedded in paraffin. Embedded tissues were cut into 6 μm thick slices, pasted onto slides, and dried. The slices were then dewaxed, washed with tap water, dehydrated with alcohol, stained with eosin for 5 min, dehydrated again, and sealed. Microscopy images were collected and analyzed.

### Immunohistochemical detection

Slices were dewaxed and dehydrated by xylene, 100, 95, 80, and 70% alcohol in turn. Citrate buffer was added and it was heated with a microwave oven. This was then cooled to room temperature and washed with PBS buffer three times. Then the steps in the streptavidin-peroxidase (SP) kit (Shanghai HengYuan Biological Technology Co., Ltd, Shanghai, China) were followed to block it. Firstly, endogenous peroxidase blocking agent was added to it for 10 min. Then, 10% goat serum blocking solution was added dropwise for 10 min. The antibodies of Claudin-1, Occludin, and ZO-1 (primary antibody) were diluted by 1:50 and then added dropwise, and a negative control was set (PBS buffer was used instead of primary antibody). It was incubated at 4°C overnight, then washed with PBS buffer three times, and a biotin-labeled goat anti-mouse IgG polymer (secondary antibody) was added dropwise. Diaminobenzidine (DAB) was added to develop color for about 8 min and differentiated after hematoxylin fine dyeing. With tap water, it turned blue and was dehydrated until it became transparent and then sealed with a film.

### 16S rRNA gene sequencing

Total genomic DNA from the jejunal feces samples (100 mg) from each piglet was extracted using the E.Z.N.A.^®^ Stool DNA Kit (D4015, Omega, Inc., USA), following the manufacturer's instructions. Corresponding primers were designed for the conserved region of the 16S rRNA nucleotide sequence. Then, the variable region (V3 + V4) of the 16S rRNA gene of intestinal microorganisms, or a specific gene fragment, was amplified by PCR forward primer 341F (5'-cctacggggcgcag-3') and reverse primer 80R (5'-gactachvggtatctatcc-3'). The PCR reaction system and conditions are shown in Supplementary materials S2, S3, respectively. PCR products were detected by 2% agarose gel electrophoresis and further purified. The purified PCR products were evaluated by an Agilent 2100 Bioanalyzer (Agilent, USA) and Illumina library quantitative kit (Kapa Biosciences, Woburn, MA, USA). The libraries were sequenced on the NovaSeq PE250 platform.

### Transcriptomics profiling

Total RNA from jejunal tissues was extracted using TRIzol reagent (Invitrogen, Carlsbad, CA, USA) and purified to obtain clean messenger RNA (mRNA) that was then decomposed into smaller fragments of 200–300 nucleotides. First-strand complementary DNA (cDNA) and second-strand cDNA were then synthesized, and the entire transcriptome profiling process was completed by LianChuan-Biotechnology Co., Ltd (Hangzhou, China). An Illumina Hiseq^TM^ high-speed sequencer was used to screen and sort sequencing data. The DESeq2 program was used to measure differentially expressed genes (DEGs), defined as a | log_2_ (fold change) | of > 1.5 and a *P*-value < 0.05. Each DEG was then subjected to the Kyoto Encyclopedia of Genes and Genomes (KEGG) and Gene Ontology (GO) enrichment analyses.

### Quantitative real-time PCR (qRT-PCR)

Total RNA from each jejunum sample was extracted using TRIzol reagent (Invitrogen, China). To verify the DEGs, Primer Premier 5.0 software was used to synthesize specific primer-related sequences of eight genes: phospholipase C gamma 2 (*PLCG2*), spleen-associated tyrosine kinase (*SYK*), phosphatidylinositol-4,5-bisphosphate 3-kinase catalytic subunit delta (*PIK3CD*), nuclear factor of activated T cells 1 (*NFATC1*) and 2 (*NFATC2*), proto-oncogene lymphocyte-specific protein tyrosine kinase (*LCK*), Bruton's tyrosine kinase (*BTK*), and B-cell linker (*BLNK*). We also designed primers to amplify three apoptosis-related genes [apoptotic peptidase activating factor 1 (*APAF1*), caspase 9 (*CASP9*), and regulator BCL2-associated X (*BAX*)] and four members of the oxidation-related *GSH-PX* gene family (*GPX1, GPX4, GPX7*, and *GPX8*). A CFX384 Touch^TM^ system (Bio-Rad, USA) was used for qRT-PCR. The reaction system of qRT-PCR was 20 μL: 10 μL of fluorescent dye, 2 μL of template diluent, 0.6 μL each of upstream and downstream primer (10 μM), and 6.8 μL of double distilled water. The expression level of β-actin was used as a standardized internal reference, and the relative mRNA expression of each gene was calculated using the 2^−ΔΔCT^ method. Primer sequences of all target genes are shown in Supplementary material S4.

### Reproducibility and coefficient of variation of kits, and statistical analysis

Reproducibility was good for all kits, and the coefficient of variation (CV; intra-assay) of all kits was ≤7.9%. All data were analyzed by IBM SPSS statistical software (v. 22.0, SPSS Inc., USA), with the pen as the experimental unit. Data related to growth performance, physiology, qRT-PCR, and Shannon, Simpson, and Chao1 indexes were all tested for conformation to the normal distribution. Subsequently, an independent samples *t*-test was conducted to examine the significance of differences. All values were expressed as the means and standard error of the mean (SEM). Differences were considered to be significant if the *P*-value was < 0.05. Asterisks represent significant differences between different groups (^*^P < 0.05, ^**^P < 0.01). If a *P*-value was > 0.05, it indicated that the difference was not significant, and it was represented by^ns^.

## Results

### Effect of transportation on the growth performance of weaned piglets

We recorded the body weight of piglets on the day of transport (age, 49 days) and 2 weeks later (age, 63 days). Analysis of the average daily weight gain for this period is shown in Table 1. Neither were there significant differences in the average weights between the CON and TSG groups of piglets at 49 days and 63 days (*P* > 0.05) nor was there any significant difference in the average daily weight gain within the 2 weeks following the transport (*P* > 0.05).

### Effect of transportation on physiological and biochemical indexes of the jejunum

Stress-related indexes in the jejunum of the two groups of piglets, including oxidative stress, are shown in Table 2. Compared to the control group, the TSG group showed significant increases in the levels of cortisol and HSP70 (*P* < 0.01), as well as HSP90 (*P* < 0.05), in the jejunum. ROS activity was also significantly increased (*P* < 0.01) in the TSG group piglets, but there was no between-group difference in GSH-Px (*P* > 0.05). Regarding the inflammatory indexes in the jejunum, the TSG group showed significant increases in the concentrations of CRP, IL-6, IL-12, and IFN-γ compared with the CON group (*P* < 0.01) (Table 2). The jejunal concentrations of tight junction proteins claudin-1, occludin, and ZO-1 were also measured, and no significant differences were detected between the TSG and CON groups (*P* > 0.05) (Table 2).

**Table 2:** Effect of transportation on physiological and biochemical indexes of jejunum^a^.

**Indexes**	**CON**	**TSG**	**SEM**	***P*-value**
Cortisol content (μg/L)	134.95	158.68	1.251	< 0.001^**^
HSP-70 content (pg/mL)	571.83	661.11	7.217	< 0.001^**^
HSP-90 content (pg/mL)	499.47	522.37	9.305	0.034^*^
GSH-Px activity (u/mL)	169.62	168.56	0.534	0.075
ROS activity (u/mL)	249.60	280.32	2.984	< 0.001^**^
CRP content (μg/L)	2,049.04	2,188.01	138.967	0.009^**^
IL-6 content (ng/L)	1,028.80	1,243.88	13.081	< 0.001^**^
IL-8 content (ng/L)	404.45	475.85	8.351	< 0.001^**^
IL-12 content (ng/L)	271.20	330.78	3.112	< 0.001^**^
IFN-γ content (pg/mL)	2123.52	2304.47	29.974	< 0.001^**^
Claudin-1 content (pg/mL)	298.37	318.58	9.237	0.069^ns^
Occludin content (pg/mL)	301.08	288.99	15.175	0.709^ns^
ZO-1 content (pg/mL)	164.77	162.18	6.723	0.444^ns^

a The values represent the means of 6 pens, with 6 piglets in each repeated pen (*n* = 36) for indexes and 6 pens in each group (*n* = 6).

SEM, Standard Error of Mean.

*p*-value = the *p*-value.

* *P* < 0.05.

** *P* < 0.01.

ns Represented no significant difference, *P* > 0.05.

Stress and reactive oxygen species indexes include Cortisol, Cortisol hormone; HSP70, Heat shock protein; HSP90, Heat shock protein; ROS, Reactive oxygen species; Inflammation indexes include CRP, C-reactive protein; IL-6, Interleukin 6; IL-12, Interleukin 12; IFN-γ, Interferon-γ.

Tight junction protein indexes include Claudin-1, Claudin-1 protein; Occluding, Occluding protein; ZO-1, zonula occudens-1 protein.

### The results of western blot and immunohistochemistry

**Figure 1:**
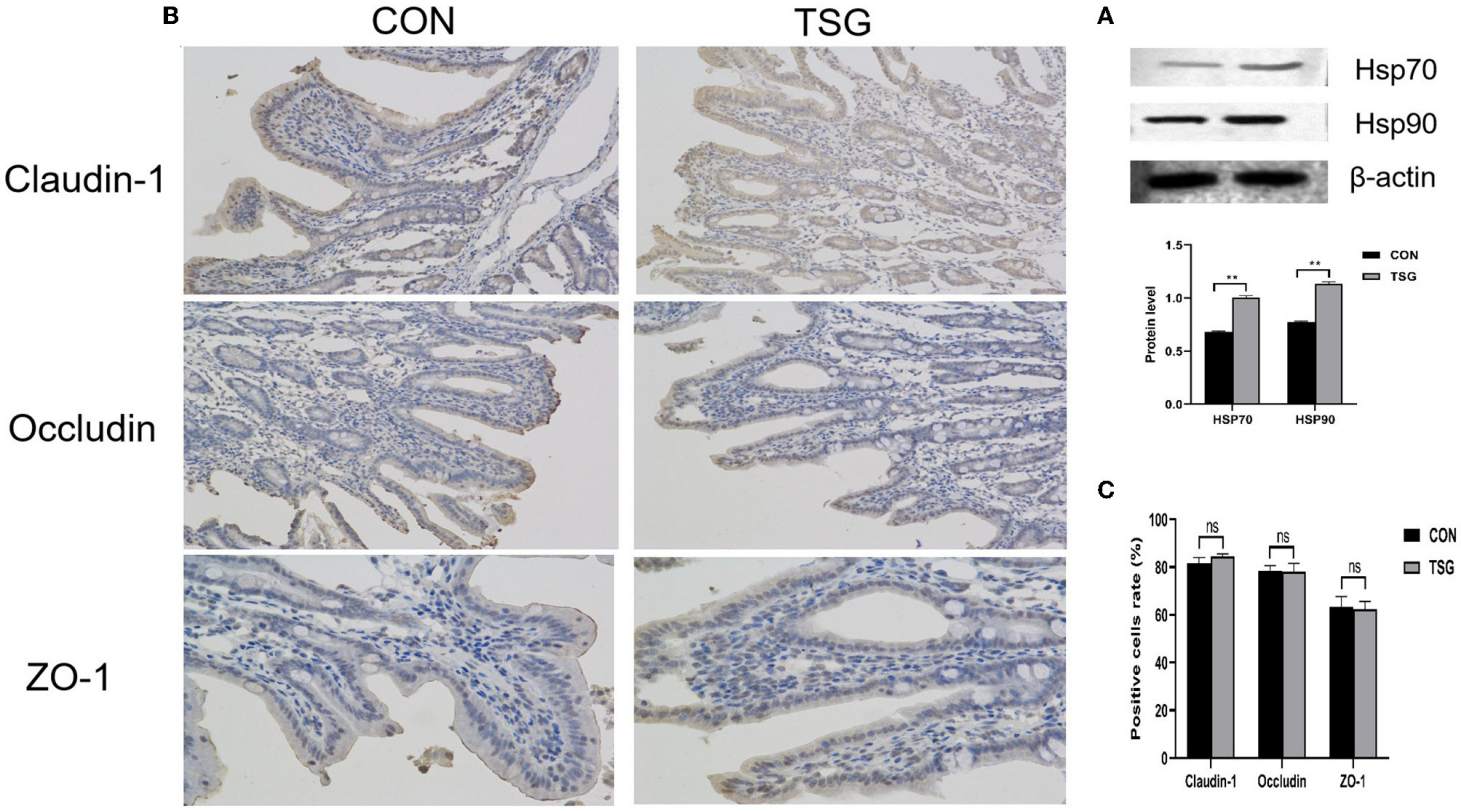
The results of western blot and immunohistochemistry. **(A)** Showed that the relative expression levels of HSP70 and HSP90 proteins increased significantly after transportation (HSP 70, **P < 0.01; HSP 90, **P < 0.01). **(B)** was the result of immunohistochemical staining of jejunum tight junction protein, and the positive signal was reddish brown. **(C)** Showed the proportion of tight junction protein (Claudin-1, Occludin, ZO-1) positive cells, and the ^ns^represented that there was no significant difference between transport group and stress group.

To improve the accuracy and visibility of the determination results of HSP proteins and tight junction proteins, we detected the HSP 70 and HSP 90 by western blot and determined the tight junction protein by immunohistochemistry. All the results are shown in Figure 1. Consistent with the results of ELISA, the protein contents of HSP70 and HSP90 were significantly expressed after transportation (HSP 70, HSP90, *P* < 0.05; Figure 1A), and the contents of the tight junction proteins did not change significantly after transportation (Claudin-1, Occludin and ZO-1, *P* > 0.05, represented by ^ns^; Figures 1B, C).

### Effect of transportation on intestinal morphology

**Figure 2:**
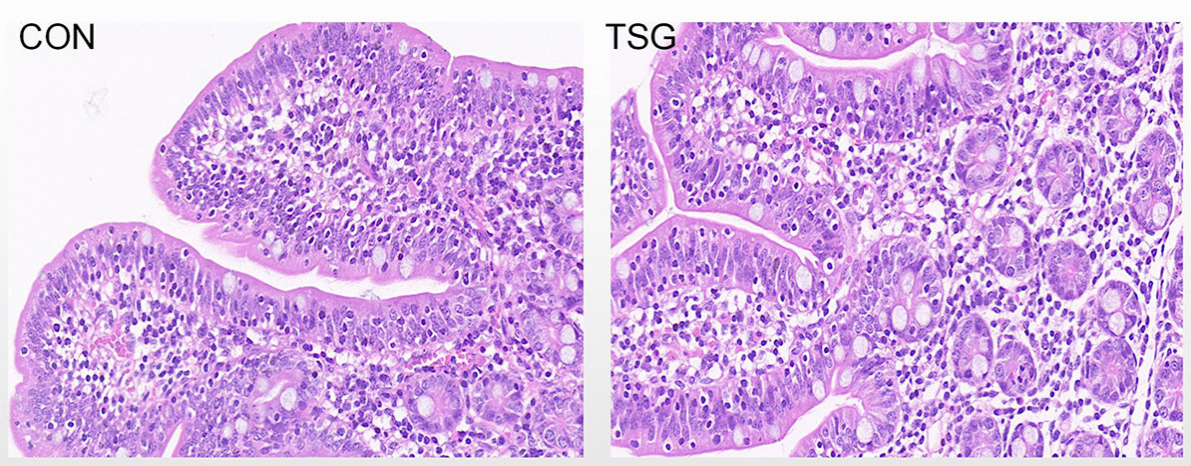
The results of intestinal morphology. The jejunum tissues taken in the experiment were intestinal segments about 4 cm after 5 cm close to the proximal end. Hematoxylin and eosin staining with original magnification × 40.

Figure 1 shows the effects of transportation on the morphology of the jejunum in piglets. In the field of view of the jejunum in the TSG group, there was mild lymphocyte infiltration of the mucosal epithelium, edema of the lamina propria, and loose connective tissue arrangement (Figure 2B). By contrast, the CON group showed a lack of lymphocyte infiltration of the mucosal epithelium, a lack of edema of the lamina propria, and tight connective tissue arrangement (Figure 2A).

### 16S rRNA: Venn diagram and alpha diversity analysis

**Figure 3:**
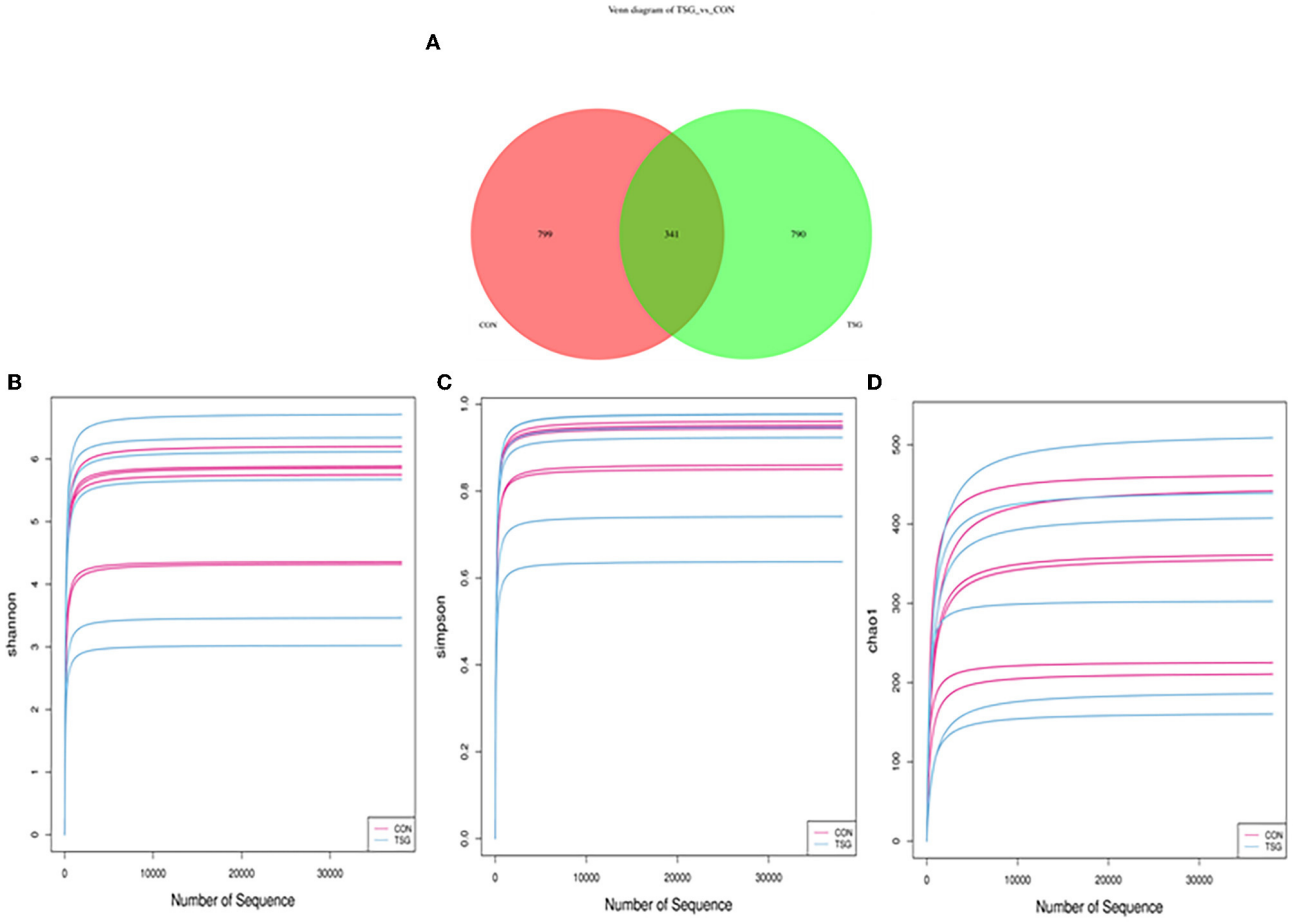
16S rRNA: Venn diagram and alpha diversity analysis. **(A)** Showed that the pink area was the unique OTUs of the control group. The green area was the unique OTUs of the TSG group. The overlapping area in the middle is common to the TSG group of con combination. In addition, **(B–D)** separately showed the dilution curve corresponding to Shannon **(B)**, Simpson **(C)**, and Chao1 index **(D)**.

As shown in Figure 3A, the total number of operational taxonomic units (OTUs) in the jejunal fecal samples in the CON group was 1,140. The TSG group had 1,131 OTUs, of which 341 were shared by the two groups, accounting for 17.67% of the total number of OTUs. There were 799 unique OTUs in the CON group (41.40% of the total OTUs) and 790 unique OTUs in the TSG group (40.93% of the total OTUs).

Figures 3B–D demonstrates that the dilution curves of all samples tended to be gentle, which may accurately reflect the species and structural diversity of microbial communities in the jejunum. Additionally, as shown in Table 3, there were no significant between-group differences in the Shannon, Simpson, and Chao1 index values of jejunal microorganisms (*P* > 0.05).

**Table 3:** Effects of transportation stress on richness and diversity of jejunum in weaned piglets^a^.

**Indexes**	**CON**	**TSG**	**SEM**	***p*-value**
Shannon	5.39	5.22	0.340	0.820^ns^
Simpson	0.92	0.86	0.304	0.420^ns^
Chao1	340.18	330.83	33.887	0.898^ns^

a The values represent the means of 6 pens, with 6 piglets in each repeated pen (*n* = 36) for indexes and 6 pens in each group (*n* = 6).

SEM, Standard Error of Mean.

*p*-value = the *p*-value.

ns Represented no significant difference, *P* > 0.05.

Shannon, Shannon index; Simpson, Simpson index; Chao 1, Chao 1 index.

### 16S rRNA: Microbial composition

**Figure 4:**
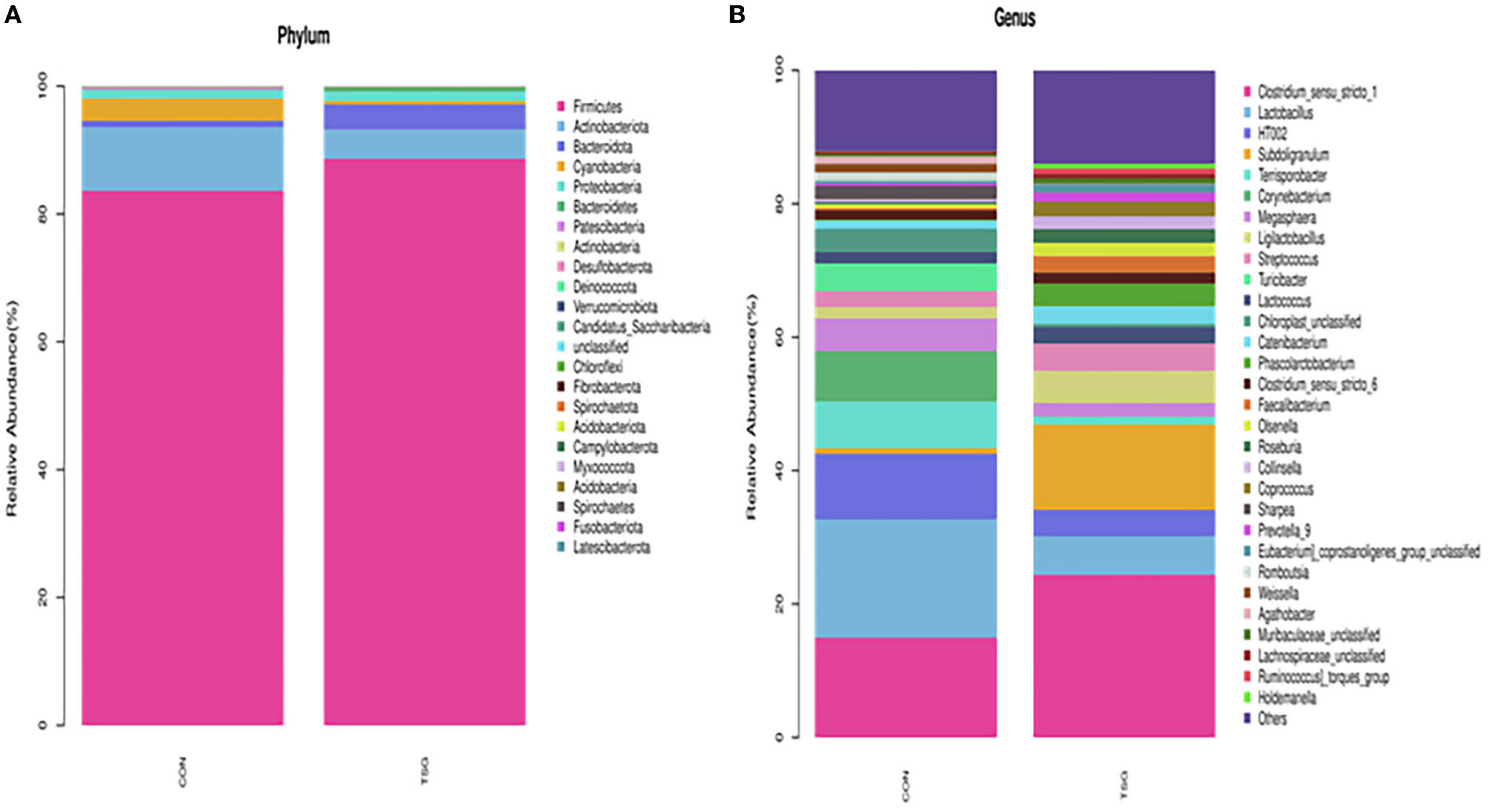
16S rRNA: Microbial composition. **(A)** Bacteria detected at the phylum level. **(B)** Bacteria detected at the genus level.

At the phylum level, 23 phyla were detected in the jejunum of the two groups (Figure 4A). The dominant phyla of both groups were Firmicutes, Actinobacteriota, Bacteroidota, and Proteobacteria. However, there was no significant difference between the CON group and the TSG group in the dominant phyla (*P* > 0.05). At the genus level, compared with the CON group, the relative abundance of *Lactobacillus* in the jejunum of the TSG group showed a downward trend, while the relative abundance of *Muribaculaceae_unclassified* showed an upward trend, but the differences were not significant (*P* > 0.05) (Figure 4B).

### Transcriptomics profiling results

**Figure 5:**
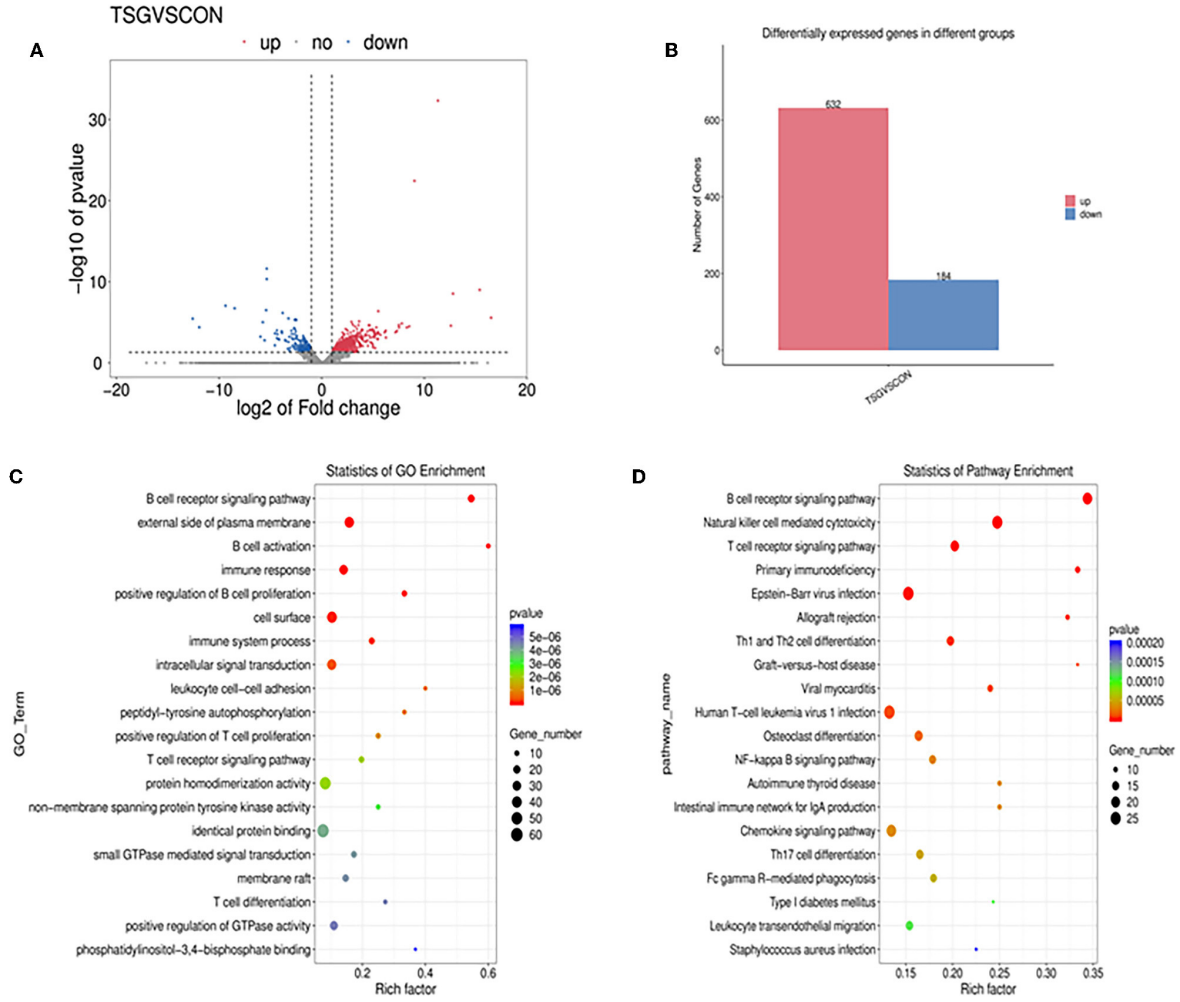
The results of the transcriptomics profiling technique. **(A)** A volcano plot of all genes differentially expressed. Red represents significantly upregulated genes, blue represents significantly down-regulated genes, and gray dots represent non-significantly differentially expressed genes. **(B)** Histogram of the number of differentially expressed genes between the transport stress group and the control group. Genes that are upregulated in red and downregulated in blue. **(C)** GO terms scatter plot of GO terms enrichment in the top 20 pathways and **(D)** KEGG pathway analysis of DEGs in the top 20 pathways. The X-axis represents the degree of enrichment. In the scatter diagram, the size of the dot represents the gene number and the color of the dot represents the *P*-value of enrichment analysis.

To study changes in gene expression in piglet jejunum caused by transportation, DEGs were detected by transcriptome sequencing. As shown in the volcano plot and bar chart, a total of 816 DEGs were identified between the two groups, of which 632 genes were upregulated and 184 genes were downregulated in the TSG group (Figures 5A, B).

To better understand the gene functions and gene products involved in transportation, a scatter plot was used to summarize the GO term enrichment in the pathway analysis. Among the top 20 GO terms (Figure 5C), the first five terms were “B cell receptor signaling pathway”, “external side of plasma membrane”, “B cell activation”, “immune response”, and “positive regulation of B cell proliferation”.

The KEGG database was used to annotate the DEGs in jejunal tissue cells to determine their biological functions. The results showed that there were 67 pathways with significant differences. Figure 5D shows the 20 most significant pathways, which included “B cell receptor signaling pathway”, “natural killer cell mediated cytotoxicity”, “T cell receptor signaling pathway”, “TH1 and TH2 cell differentiation”, and “NF-kappa B signaling pathway”.

### Quantitative real-time PCR (qRT-PCR)

To ensure the accuracy of our transcriptomics analysis, we verified the variation tendency of expression of eight genes selected from each group using qRT-PCR. As shown in Table 4, the expression levels of *PLCG2, SYK, PIK3CD, NFATC1, NFATC2, LCK, BTK*, and *BLNK* were all significantly upregulated in the jejunum of the TSG group compared with those in the CON group (*P* < 0.05). These results were consistent with the transcriptomics profiling results, indicating the strength of our transcriptomics analysis. Additionally, there were no differences in the expression of three apoptosis-related genes (*APAF1, CASP9*, and *BAX*) and four members of the oxidation-related GSH-PX family (*GPX1, GPX4, GPX7*, and *GPX8*) (*P* > 0.05) (Table 4).

**Table 4:** Validation of transcriptomics sequencing using qRT-PCR.

**Genes**	**CON vs. TSG difference multiple**		**CON vs. TSG** ***P*****-value**	
	**Transcriptome** ^a^	**qRT-PCR** ^b^	**Transcriptome** ^a^	**qRT-PCR** ^b^
PLCG2	1.93	2.85	0.021^*^	< 0.001^**^
SYK	3.93	3.32	< 0.001^**^	< 0.001^**^
PIK3CD	2.83	3.12	< 0.001^**^	< 0.001^**^
NFATC1	2.67	3.05	< 0.001^**^	< 0.001^**^
NFATC2	2.36	3.53	< 0.001^**^	< 0.001^**^
LCK	2	2.63	< 0.001^**^	< 0.001^**^
BTK	3.08	2.78	< 0.001^**^	< 0.001^**^
BLNK	1.61	1.86	0.011^*^	< 0.001^**^
**Genes**	**CON vs. TSG difference multiple qRT-PCR** ^b^		**SEM**	***P*** **-value**
APAF1	1.20		0.095^ns^	0.06^ns^
CASP9	1.05		0.082^ns^	0.544^ns^
BAX	0.92		0.165^ns^	0.649^ns^
GPX1	0.85		0.125^ns^	0.263^ns^
GPX4	0.87		0.119^ns^	0.316^ns^
GPX7	1.07		0.061^ns^	0.224^ns^
GPX8	1.13		0.074^ns^	0.107^ns^

a The values represent the means of 6 pens, with 6 piglets in each repeated pen (*n* = 36) for transcriptome and 6 pens in each group, but it was a mixture of piglet jejunum samples from two pens (*n* = 3).

b The values represent the means of 6 pens, with 6 piglets in each repeated pen (*n* = 36) for qRT-PCR and 6 pens in each group, but it was a mixture of piglet jejunum samples from two pens (*n* = 3).

SEM, Standard Error of Mean.

*p*-value = the *p*-value.

* *P* < 0.05.

** *P* < 0.01.

ns Represented no significant difference, *P* > 0.05.

Difference Genes were designed to measure differentially expressed genes when the | log2 (Fold Change) | greater than 1.5 and a *P*-value of less than 0.05. The difference between multiple and *P*-values of transcriptome genes was shown according to the transcriptome results. The difference multiple and the *P*-value of qRT-PCR were displayed as relative mRNA expression levels. The relative expression level of mRNA was calculated based on the 2^−Δ*ΔCT*^ method.

## Discussion

Animals are easily affected by issues that arise in the transportation environment, such as hunger, water shortage, fright, road conditions, mixing of different pigs, and changes in loading density and temperature, all of which may cause great suffering. Exposure to such stressful environments leads to the release of cortisol and an increase in HSPs, which are widely regarded as physiological evidence of acute stress (17–19). In this study, we found that the levels of cortisol and HSP70 and HSP90 proteins were significantly increased in the jejunum of piglets that were subjected to the stress of road transportation lasting only 2 h. These results were consistent with a study by Rioja-Lang et al. (2), who found that short-distance transportation (≤2 h) can cause an increase in cortisol levels and trigger the stress response of the body (2). In a study of long-distance transportation, Zhang et al. (20) also found that pigs transported for 5 h showed increases in cortisol levels and HSPs (20). Additionally, as an essential organ for digestion, absorption, and nutrient metabolism, the small intestine can easily become the target of adverse reactions to stressors such as transportation (16, 21). Furthermore, intestinal damage also affects the feed intake of piglets, impacting their growth performance. However, our study showed that short-distance transportation did not change the growth performance of piglets within 2 weeks. Sutherland et al. (6) pointed out that, although transportation may adversely affect the performance of weaned piglets, the risk is related to transportation time (6, 22). In our road transportation experiment with piglets, the lack of intestinal damage and change in growth performance within 14 days may have been related to the short transportation time.

Transportation can also induce inflammatory reactions in animals (23). During transportation, CRP and ILs were secreted in large quantities as responders to the inflammatory response. This was consistent with a study of long-distance transportation reported by Zou et al. (15), which showed that 5 h of transportation increased inflammatory factors in the intestine (15). Our short-distance transportation study showed similar results. The concentrations of CRP, IL-6, IL-8, IL-12, and IFN-γ in the jejunum were significantly increased in the TSG group of piglets, indicating that 2 h of road transportation triggered an inflammatory reaction in the jejunum. Additionally, transcriptomics analysis also showed that genes related to inflammation were upregulated in the TSG group, including *PLCG2, SYK, PIK3CD, NFATC1, NFATC2, LCK, BTK*, and *BLNK*. Interestingly, these genes are all part of the B-cell receptor signaling pathway, which was consistent with the KEGG enrichment results showing that transportation mainly activated inflammation-related pathways. The B-cell receptor signaling pathway mainly regulates B-cell development and participates in the inflammatory response, including the regulation of inflammatory factors, the immune response, and immunoglobulin production. Additionally, the mucosal barrier of intestinal epithelial cells can be easily damaged under transportation stress, during which epithelial cells of the intestinal villi rupture and fall off, thereby inducing morphological changes in intestinal tissue (24). In our TSG group piglets, there was mild lymphocyte infiltration, edema of the lamina propria, and loose arrangement of connective tissue in the jejunum. We speculated that intestinal inflammation triggered by transportation stress may have led to the intestinal damage indicated by these morphological changes.

Studies have shown that acute stress, weaning stress, and chronic stress can all increase the level of ROS in piglets (25). ROS may trigger intestinal damage and dysfunction in stressed animals (26). Excessive ROS destroys cytoskeleton proteins and the intestinal barrier, increases intestinal permeability, and ultimately causes intestinal dysfunction (27). The intestinal barrier is an important doorway between the intestine and the outside world, and the tight junction proteins, located at the top of the outer membrane of intestinal epithelial cells, comprise the most important intercellular junction connecting intestinal epithelial cells (28). Zou et al. (15) showed that 5 h of transportation increased ROS and decreased the expression of tight junction proteins, leading to oxidative damage of the jejunal barrier in pigs (15). By contrast, our study showed that short-distance transportation lasting 2 h caused an increase in ROS but had no significant effect on claudin-1, occludin, or ZO-1 levels in the jejunum of piglets. We speculate that the increase in ROS during short-distance transportation was not high enough to cause oxidative damage to jejunum cells and damage the jejunal barrier. This possibility was also supported by the transcriptomics analysis, which showed no significant between-group differences in the expression of apoptosis-related genes such as *APAF1, CASP9*, and *BAX*, among which *CASP9* plays a major role in apoptosis control (29). These results suggested that short-distance transportation did not activate the apoptosis pathway in the jejunum. Similarly, there were no significant differences in genes related to oxidative stress, including GSH-PX family members *GPX1, GPX4, GPX7*, and *GPX8*. GSH-PX is a key enzyme antioxidant that protects cells from oxidative damage by reducing the rising state of oxidative stress, eliminating the toxicity of peroxide, and transforming it into a non-toxic substance (30). Additionally, ROS can also act as a signal molecule to transcriptionally activate the apoptosis signaling pathways, promote intestinal inflammatory response, and start the intestinal cell apoptosis program (31). Our results showed that the increase in ROS following short-distance transportation mainly caused inflammation but did not lead to oxidative damage or apoptosis pathway activation in the jejunum of piglets. By contrast, in the Zou et al. (15) study, 5 h of transportation increased the expression of inflammatory cytokines in the pig intestinal tract, leading to changes in apoptosis-related genes and pathways (15). We speculate that the excessive degree of ROS generation occurring over 5 h in their study directly led to intestinal injury, whereas the increase in ROS in our study did not meet the threshold for such an injury.

The intestinal microbiome plays an important role in maintaining the integrity of intestinal barrier function, but the structure of the intestinal microbiome gets altered under stress (32). In this study, the microbial composition of jejunal fecal matter was evaluated using high-throughput sequencing technology. The dilution curves of the sequencing results of each sample tended to be flat, indicating that the results accurately reflected the species and structural diversity of microbial communities in the jejunum. Alpha diversity metrics include the Shannon and Simpson indexes, which reflect the diversity of a microbial community, and the Chao1 index, which reflects the richness of a microbial community. There were no significant differences between the CON and TSG groups in the Shannon, Simpson, or Chao1 indexes of jejunal microorganisms, indicating that 2 h of short-distance transportation had no effect on microbial diversity and richness in the jejunum. Unfortunately, there are very few studies on transport-related effects on microbial diversity of pig intestines with which to compare our results. Our sequencing results showed that short-distance transportation had no significant effect on the dominant phyla of jejunum microorganisms in piglets. However, at the genus level, the relative abundance of *Lactobacillus* showed a downward trend, while that of *Muribaculaceae_unclassified* showed an upward trend, in the jejunum of the TSG group. *Lactobacillus* has been related to improved stress response in humans (33). As a probiotic, *Lactobacillus* can scavenge ROS and free radicals, reducing their damage to organisms and cells and inhibiting stress and inflammatory reactions (34, 35). By contrast, Gram-negative *Muribaculaceae_unclassified* is a common pathogenic bacteria. Reports by Campbell et al. (36) and Dang and Kim (37) describing the transportation of pigs as leading to an increase in the number of harmful bacteria in the intestine (36, 37) is consistent with the results of our study.

## Conclusions

The 2-h short-distance road transportation experiment in this study increased stress levels and caused intestinal inflammation in the jejunum of piglets. It also decreased beneficial microorganisms and increased harmful microorganisms in the jejunum, although not significantly. Interestingly, although the short-distance transportation increased ROS generation in the jejunum, it did not lead to oxidative damage or apoptosis pathway activation in the jejunum. Our findings provide valuable systematic data on the influence of short-distance transportation on piglet intestines, providing an accurate reference for improving the welfare of piglets during short-distance transportation.

## Data availability statement

The datasets presented in this study can be found in online repositories. The name of the repository and accession numbers can be found below: NCBI Sequence Read Archive; PRJNA934118 and PRJNA935285.

## Ethics statement

The animal study was reviewed and approved by Animal Protection and Utilization Committee of Northeast Agricultural University (NEAUEC20200346).

## Author contributions

HL: conceptualization, methodology, validation, writing—review and editing, and supervision. QF: conceptualization, methodology, software, formal analysis, data curation, writing—original draft, and visualization. XY: software, formal analysis, data curation, writing—review and editing, and visualization. SZ: resources and visualization. YY: resources and data curation. XZ: resources. QH and WJ: data curation. All authors contributed to the article and approved the submitted version.

## References

[1] Lewis NJ. Transport of early weaned piglets. Appl Anim Behav Sci. (2008) 110:128–35. 10.1016/j.applanim.2007.03.027

[2] Rioja-Lang FC Brown JA Brockhoff EJ Faucitano L. A review of swine transportation research on priority welfare issues: a Canadian perspective. Front Vet Sci. (2019) 6:36. 10.3389/fvets.2019.00036 30854374 PMC6395376

[3] Peeters E Driessen B Steegmans R Henot D Geers R. Effect of supplemental tryptophan, vitamin E, and a herbal product on responses by pigs to vibration[J]. J Anim Sci. (2004) 82:2410–20. 10.2527/2004.8282410x 15318742

[4] Roldan-Santiago P Trujillo-Ortega M Borderas-Tordesillas F Martínez-Rodríguez R Mora-Medina P Flores-Peinado S. Physiometabolic responses to road transport in weaned piglets for a short period and the effects of straw bedding. Anim Sci J. (2015) 86:563–71. 10.1111/asj.12324 25496132

[5] Garcia A Sutherland M Pirner G Picinin G May M Backus B. Impact of providing feed and/or water on performance, physiology, and behavior of weaned pigs during a 32-h transport. Animals. (2016) 6:31. 10.3390/ani6050031 27153096 PMC4880848

[6] Sutherland MA Backus BL McGlone JJ. Effects of transport at weaning on the behavior, physiology and performance of pigs. Animals. (2014) 4:657–69. 10.3390/ani4040657 26479005 PMC4494433

[7] Nielsen BL Dybkjaer L Herskin MS. Road transport of farm animals: effects of journey duration on animal welfare. Animal. (2011) 5:415–27. 10.1017/S1751731110001989 22445408

[8] Pluske JR Miller DW Sterndale SO. Associations between gastrointestinal-tract function and the stress response after weaning in pigs. Anim Prod Sci. (2019) 59:2015–22. 10.1071/AN19279

[9] Radwan MA El-Gendy KS Gad AF. Oxidtive stress biomarkers in the digestive gland of theba pisana exposed to heavy metals. Arch Environ Con Tox. (2010) 58:823–35. 10.1007/s00244-009-9380-1 19705050

[10] Reuter S Gupta SC Chaturvedi MM Aggarwal BB. Oxidative stress, inflammation, and cancer: how are they linked? Free Radic Biol Med. (2010) 49:1603–16. 10.1016/j.freeradbiomed.2010.09.006 20840865 PMC2990475

[11] Buchholz BM Kaczorowski DJ Sugimoto R Yang R Wang Y Billiar TR. Hydrogen inhalation ameliorates oxidative stress in transplantation induced intestinal graft injury. Am J Transplant. (2008) 8:2015–24. 10.1111/j.1600-6143.2008.02359.x 18727697

[12] Tian R Tan J-T Wang R-L Xie H Qian Y-B Yu K-L. The role of intestinal mucosa oxidative stress in gut barrier dysfunction of severe acute pancreatitis. Eur Rev Med Pharmacol Sci. (2013) 17:349–55. 23426538

[13] Handa O Majima A Onozawa Y Horie H Uehara Y Fukui A. The role of mitochondria-derived reactive oxygen species in the pathogenesis of non-steroidal anti-inflammatory drug-induced small intestinal injury. Free Radic. Res. (2014) 48:1095–9. 10.3109/10715762.2014.928411 24870068

[14] Perry E Cross T-WL Francis JM Holscher HD Clark SD Swanson KS. Effect of road transport on the equine cecal microbiota. J Equine Vet Sci. (2018) 68:12–20. 10.1016/j.jevs.2018.04.004 31256882

[15] Zou Y Wei HK Xiang Q-H Wang J Zhou Y-F Peng J. Protective effect of quercetin on pig intestinal integrity after transport stress is associated with regulation oxidative status and inflammation. J Vet Med Sci. (2016) 78:1487–94. 10.1292/jvms.16-0090 27301842 PMC5059377

[16] Zhong X Zhang XH Li XM Zhou YM Li W Huang XX. Intestinal growth and morphology is associated with the increase in heat shock protein 70 expression in weaning piglets through supplementation with glutamine. J Anim Sci. (2011) 89:3634–42. 10.2527/jas.2010-3751 21705630

[17] Goddard PJ Keay G Grigor PN. Lactate dehydrogenase quantification and isoenzyme distribution in physiological response to stress in red deer (Cervus elaphus). Res Vet Sci. (1997) 63:119–22. 10.1016/S0034-5288(97)90003-5 9429243

[18] Garrido C Ottavi P Fromentin A Hammann A Arrigo AP Chauffert B. HSP27 as a mediator of confluence-dependent resistance to cell death induced by anticancer drugs. Cancer Res. (1997) 57:2661–7. 9205074

[19] Choi YM Jung KC Choe JH Kim BC. Effects of muscle cortisol concentration on muscle fiber characteristics, pork quality, and sensory quality of cooked pork. Meat Sci. (2012) 94:490–8. 10.1016/j.meatsci.2012.03.003 22498136

[20] Zhang T Zhou YF Zou Y Hu X. Effects of dietary oregano essential oil supplementation on the stress response, antioxidative capacity, and HSPs mRNA expression of transported pigs. Livest Sci. (2015) 180:143–9. 10.1016/j.livsci.2015.05.037

[21] Hao Y Gu XH Wang XL. Overexpression of heat shock protein 70 and its relationship to intestine under acute heat stress in broilers: 1. Intestinal structure and digestive function. Poult Sci. (2012) 91:781–9. 10.3382/ps.2011-01627 22399715

[22] Wamnes S Lewis NJ Berry RJ. The behaviour of early-weaned piglets following transport: Effect of season and weaning weight. Can J Anim Sci. (2008) 88:357–67. 10.4141/CJAS07083

[23] Wan C Yin P Xu X Liu M He S Song S. Effect of simulated transport stress on the rat small intestine: a morphological and gene expression study. Res Vet Sci. (2014) 96:355–64. 10.1016/j.rvsc.2014.01.008 24560020

[24] Cuppoletti J Blikslager AT Chakrabarti J Nighot PK Malinowska DH. Contrasting effects of linaclotide and lubicrostone on restitution of epithelial cell barrier properties and cellular homeostasis after exposure to cell stressors. BMC Pharmacol. (2012) 12:3. 10.1186/1471-2210-12-3 22553939 PMC3403872

[25] Wei HK Xue HX Zhou Z Peng J. A carvacrol–thymol blend decreased intestinal oxidative stress and influenced selected microbes without changing the messenger RNA levels of tight junction proteins in jejunal mucosa of weaning piglets. Animal. (2017) 2:193–201. 10.1017/S1751731116001397 27416730

[26] Zhu LH Zhao KL Chen XL Xu JX. Impact of weaning and an antioxidant blend on intestinal barrier function and antioxidant status in pigs. J Anim Sci. (2012) 90:2581–9. 10.2527/jas.2011-4444 22896732

[27] Bhattacharyya A Chattopadhyay R Mitra S Crowe SE. Oxidative stress: an essential factor in the pathogenesis of gastrointestinal mucosal diseases. Physiol Rev. (2014) 94:329–54. 10.1152/physrev.00040.2012 24692350 PMC4044300

[28] Turner JR. Intestinal mucosal barrier function in health and disease. Nat Rev Immunol. (2009) 9:799–809. 10.1038/nri2653 19855405

[29] Zhou X Zeng W Li H Chen H Wei G Yang X. Rare mutations inapoptosis related genes APAF1, CASP9, and CASP3 contribute to human neural tube defects. Cell Death Dis. (2018) 9. 10.1038/s41419-017-0096-2 29352212 PMC5833651

[30] Altuhafi M Altun M Hadwan MH. The correlation between selenium-dependent glutathione peroxidase activity and oxidant/antioxidant balance in sera of diabetic patients with nephropathy. Rep Biochem Mol Biol. (2021) 10:164–72. 10.52547/rbmb.10.2.164 34604406 PMC8480288

[31] Mittal M Siddiqui MR Tran K Reddy SP Malik AB. Reactive oxygen species in inflammation and tissue injury. Antioxid Redox Signal. (2014) 20:1126–67. 10.1089/ars.2012.5149 23991888 PMC3929010

[32] Aguilera M Vergara P Martínez V. Stress and antibiotics alter luminal and wall-adhered microbiota and enhance the local expression of visceral sensory-related systems in mice. Neurogastroenterol Motil. (2013) 25:e515–29. 10.1111/nmo.12154 23711047

[33] Aizawa E Tsuji H Asahara T Takahashi T Teraishi T Yoshida S. Possible association of Bifidobacterium and Lactobacillus in the gut microbiota of patients with major depressive disorder. J Affect Disord. (2016) 202:254–7. 10.1016/j.jad.2016.05.038 27288567

[34] Murtaza N Baboota RK Jagtap S Singh DP Khare P Sarma SM. Finger millet bran supplementation alleviates obesity-induced oxidative stress, inflammation and gut microbial derangements in high-fat diet-fed mice. Br J Nutr. (2014) 112:1447–58. 10.1017/S0007114514002396 25234097

[35] Xin J Zeng D Wang H Ni X Yi D Pan K. Preventing non-alcoholic fatty liver disease through Lactobacillus johnsonii BS15 by attenuating inflammation and mitochondrial injury and improving gut environment in obese mice. Appl Microbiol Biotechnol. (2014) 98:6817–29. 10.1007/s00253-014-5752-1 24811405

[36] Campbell JM Crenshaw JD Polo J. The biological stress of early weaned piglets. J Anim Sci Biotechnol. (2013) 4:1–4. 10.1186/2049-1891-4-19 23631414 PMC3651348

[37] Dang DX Kim IH. The effects of road transportation with or without homeopathic remedy supplementation on growth performance, apparent nutrient digestibility, fecal microbiota, and serum cortisol and superoxide dismutase levels in growing pigs. J Anim Sci. (2021) 99:1–7. 10.1093/jas/skab077 33693792 PMC8051841

