# Supplementary material for: Effects of short-distance transportation on physiological indexes, intestinal morphology, microbial community, and the transcriptome of the jejunum in weaned piglets
Source: Front Vet Sci. 2023 Apr 12;10:1148941. doi: 10.3389/fvets.2023.1148941 (PMC10130522; doi:10.3389/fvets.2023.1148941)
Supplement: Supplementary file 1 [file Data_Sheet_1.docx]

**Effects of short distance transportation on physiological and biochemical indexes, microbial community and transcriptome of jejunum in Weaned Piglets**

**Qin. Fu^1a^, Xuesong. Yang^1a^, Sitong. Zhou^a^, Xiaohong. Zhang^a^, Qi. Han^a^, Wenbo. Ji^a^, Honggui. Liu^a,b,^***

*^a^ College of Animal Science and Technology, Northeast Agricultural University,* *Changjiang Road No. 150030 Harbin, Heilongjiang, P.R. China; ^b^ Key Laboratory of Swine Facilities Engineering, Ministry of Agriculture and Rural Affairs, Changjiang Road No. 150030 Harbin, Heilongjiang, P.R. China*

^*^ Corresponding author: H.G Liu. Email: Hg.Liu@neau.edu.cn (H. Liu)

^1^ represents these authors have equal contribution to this work.

**Supplementary material**

**Supplementary S1 Nutritional composition of Creep feed and Nursery diet**

| Creep feed Ingredients | Contents (%) | Creep feed Nutrient levels | Contents (%) |
| --- | --- | --- | --- |
| Extruded corn | 46.30 | Metabolizable energy (MJ/Kg) | 14.23 |
| Soybean meal | 8.00 | Crude protein | 20.10 |
| Extruded soynean | 12.00 | Available phosphorus | 0.56 |
| Wheat hydrolyzed protein | 4.00 | Calcium | 0.89 |
| Whey poeder (3%) | 8.00 | Digestible lysine | 1.55 |
| Lactose | 8.00 | Digestible tryptophan | 0.25 |
| Dried porcine solules | 3.00 | Digestible methionine | 0.45 |
| White fish meal | 5.00 | Digestible threonine | 0.99 |
| Vegetable oil | 1.50 |  |  |
| Limestone | 0.70 |  |  |
| CaHPO_4_ | 1.50 |  |  |
| Premix^1^ | 2.00 |  |  |

| Nursery diet Ingredients | Contents (%) | Nursery diet Nutrient levels | Contents (%) |
| --- | --- | --- | --- |
| Corn | 70.00 | Metabolizable energy (MJ/Kg) | 13.37 |
| Soybean meal | 18.00 | Crude protein | 17.00 |
| Extruded soynean | 4.40 | Available phosphorus | 0.43 |
| Fish meal | 3.00 | Calcium | 0.84 |
| Limestone | 0.90 | Digestible lysine | 1.00 |
| CaHPO_4_ | 1.40 | Digestible tryptophan | 0.16 |
| NaCl | 0.30 | Digestible methionine | 0.24 |
| Premix^1^ | 2.00 | Digestible threonine | 0.64 |
| Total | 100.00 | NaCl | 0.45 |

^1^The premix provived the following per kilogram of diet: VA 8000 IU, VD_3_ 1228 IU, VE 151 IU, VK_3_ 3.1mg, VB_6_ 1.2 mg, VB_12_ 0.03 mg, calcium pantothenate 13.4 mg, choline chloride 500 mg, biotin 0.11 mg, niacin 25 mg, folic acid 0.68 mg, Fe 120 mg, Cu 10 mg, Zn 130 mg, Mn 100 mg, I 0.3 mg, Se 0.3 mg.

**Supplementary S2 PCR reaction system**

| PCR reaction components | PCR reaction system |
| --- | --- |
| Phusion Hot start flex 2X Master Mix | 12.5 ul |
| Forward Primer | 2.5 ul |
| Reverse Primer | 2.5 ul |
| Template DNA | 50 ng |
| Add ddH2O to | 25 ul |

**Supplementary S3 PCR reaction conditions**

| PCR reaction temperature | PCR reaction time | Cyclic number |
| --- | --- | --- |
| 98℃ | 30s | - |
| 98℃ | 10s | - |
| 54℃ | 30s | 35cycles |
| 72℃ | 45s | - |
| 72℃ | 10min | - |
| 4℃ | ∞ | - |

**Supplementary S4 Gene-specific primers used in qRT-PCR**

| Gene | Forward Primer（Five，-Three，） | Reverse Primer（Five，-Three，） |
| --- | --- | --- |
| PLCG2 | GAGCAAGACGGCGGACAAGATC | GAAGTTGACCAGCGGCAGGATG |
| SYK | AAGCCCAGAGAGAAGCCTTACCC | CAGCCATCTCCATAACCAGCATCC |
| PIK3CD | TCAACAAGGATGCTCTGCTCAACTG | CAGGATGGCATAGGCTCGTTCAC |
| NFATC1 | ACATCCGACTTCCCACCTGAGG | CTGCCGTCCCGATGAACAACTG |
| NFATC2 | GCAGAACTTGGACCAGACCTACTTG | CTGTGGACGGTGTCTTGCTGATG |
| LCK | CGACCAGAACCAAGGAGAAGTGATG | CTTCGTGTGCCCGTTGTAGTACC |
| BTK | AAGCCTCTTCCTCCCACACCTG | GCCTCCTTCTTTGCCCTCTTGC |
| BLNK | CTCAAGTCCCACCCAAACCCAAAG | GCGTTCGGCAGGTATAGGTCTTTC |
| APAF1 | TACCCTGTTGGCGACTGGAGATG | AGACTGGAGCACACGAATGAAGAAG |
| CASP9 | CATTGAGACCCTGGATGGCGTTC | CCCTTTCACTGAGACAGCATTGGAG |
| BAX | TTGCTTCAGGGTTTCATCCAGGATC | AGCCGATCTCGAAGGAAGTCCAG |
| GPX1 | CACGCTCGGTGTATGCCTTCTC | CGCCATTCACCTCACACTTCTCG |
| GPX4 | GAGGCAAGACGGAGGTAAACTACAC | TAGCACGGCAGGTCCTTCTCTATG |
| GPX7 | GCAGGACTTCTACGACTTCAAGGC | CCACATTCACCACAAGGGACACC |
| GPX8 | GCAAAAGGAAGAACGGTTTCTCTGG | CTCAGGGTTGACCAGATACTTCCAG |
| β-actin | AATCCTGCGGCATCCACGAAAC | CAGCACCGTGTTGGCGTAGAG |
